# Case report: migration of a broken screw to the knee joint after hardware failure following closing wedge distal femoral osteotomy

**DOI:** 10.1186/s12891-019-2505-4

**Published:** 2019-03-20

**Authors:** Yong Seuk Lee, Seung Hoon Lee, Eui Soo Lee, Teck Siong Fong

**Affiliations:** 1 Department of Orthopaedic Surgery, Seoul National University College of Medicine, Seoul National University Bundang Hospital, 166 Gumi-ro, Bundang-gu, Seongnam-si, Gyeonggi-do 463-707 South Korea; 2 Veterans Hospital, Seoul, Korea; 3 Department of Orthopaedic Surgery, Putrajaya Hospital, Putrajaya, Malaysia

**Keywords:** Valgus osteoarthritis, Distal femoral osteotomy, Hardware failure, Knee joint

## Abstract

**Background:**

We report a case of hardware failure after distal femoral osteotomy (DFO) with a broken screw pulled out from the locking hole and positioned within the knee joint.

**Case presentation:**

A 57-year-old man presented to our orthopedic outpatient department with 3-months history of an unusual painful swelling at the operated area following DFO. The leakage of joint fluid from the penetrated suprapatellar pouch was assumed to be the reason for this complication.

**Conclusions:**

The overall aim of this case report is to provide a lesson to budding surgeons who might experience a similar situation that cannot be easily explained, like the unexpected complication in the present case.

## Background

The common complications of osteotomies around the knee are cortical hinge fracture, unexpected loss of correction or overcorrection, hardware failures, regional pain syndrome, delayed union or nonunion, neurovascular injury, and infection [1–4]. We describe a case of migration of a broken screw to the knee joint 3 months after closing wedge distal femoral osteotomy (DFO), secondary to hardware failure. The overall aim of this report is to avoid similar complication in the future through modification of the surgical technique.

## Case presentation

**Fig. 1:**
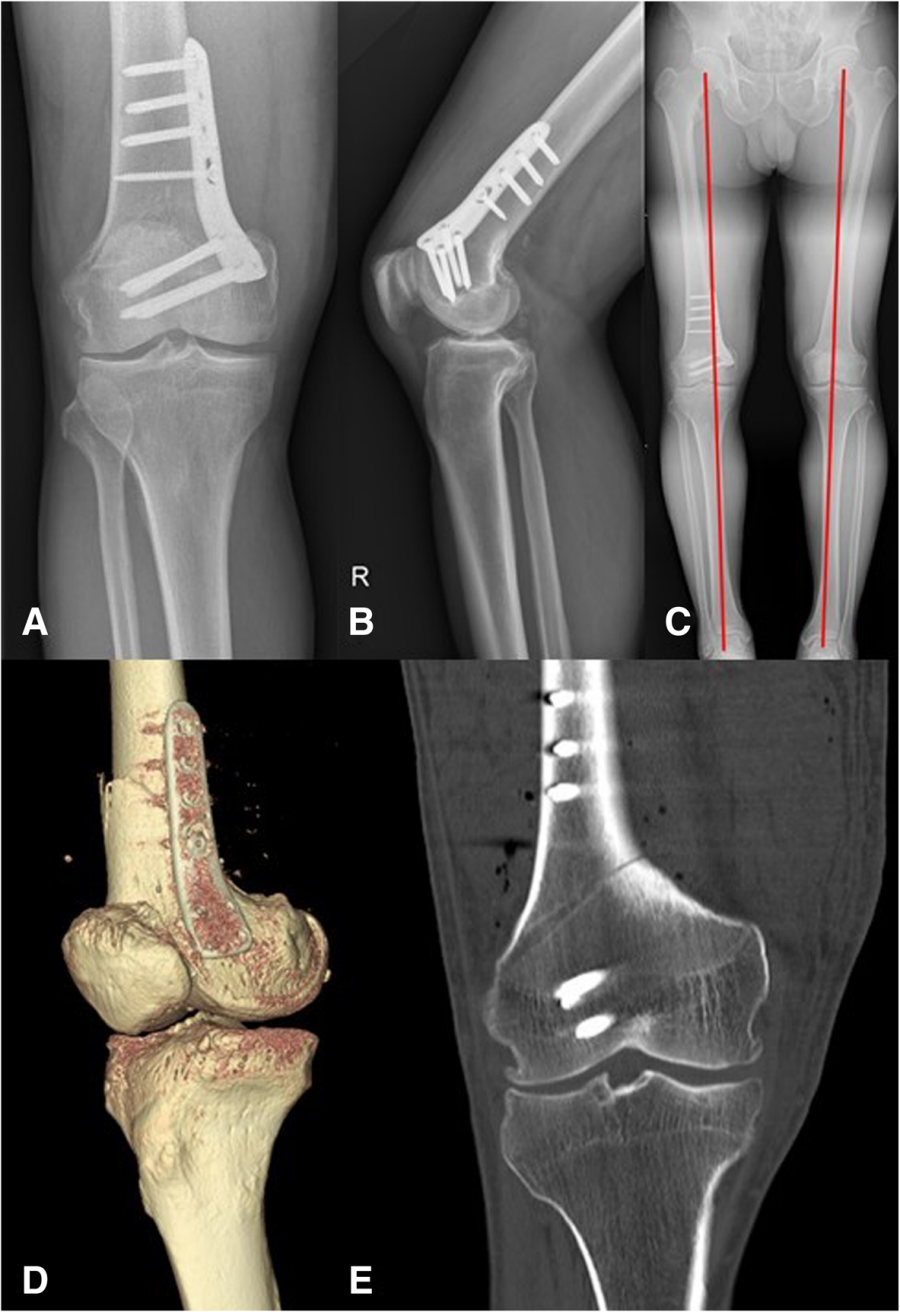
Immediate postoperative status: AP view of radiograph (**a**); lateral view of radiograph (**b**); postoperative corrected alignment (**c**); 3D reconstruction CT image shows that the plate is placed slightly frontal (**d**); and postoperative CT scan (**e**)

A 57-year-old man (weight, 89.3 kg; height, 175 cm; body mass index, 29.16 kg/m^2^) initially presented to our outpatient clinic with complaints of lateral knee pain. Radiographs revealed lateral compartment osteoarthritis and valgus deformity of the knee joint. He underwent a closing wedge DFO (Fig. 1). The target alignment was adjusted to the contralateral limb, and biplanar osteotomy was performed. Fixation was performed using a locking plate (Ohtofix®, DFO Plate, Hwaseong, South Korea). A bicortical lag screw was inserted initially at the combination hole for indirect reduction of the shaft. Subsequent self-tapping locking screws were inserted using a power driver after predrilling the screw holes and measuring with a depth gauge. Final manual tightening of the locking screws were performed in sequence with a torque-limiting screw driver. The rehabilitation protocol was routine, and tolerable weight bearing with crutches was performed after postoperative 1 week.

**Fig. 2:**
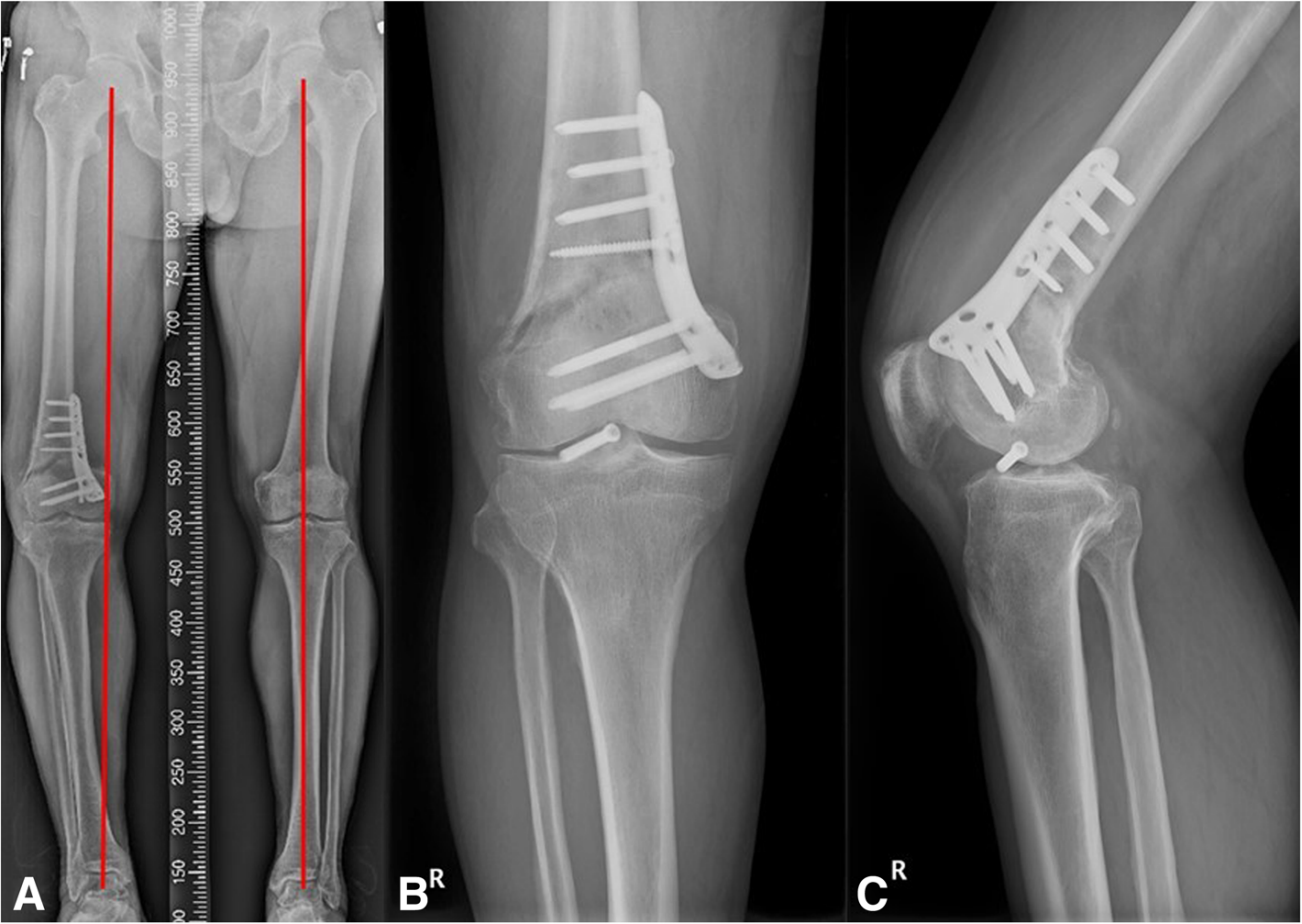
Postoperative 1-month status: collapsed varus alignment and broken distal screw (**a**); AP and lateral view of radiograph shows migration of the broken screw to the knee joint (**b**, **c**)

During follow up, he complained of an unusual painful swelling at the operated area. Aspiration was performed at postoperative 2 weeks, and 50 cc of blood was aspirated. At postoperative 1 month, he visited the emergency department because of recurrent painful swelling. More than 50 cc of joint fluid mixed with blood was aspirated. Radiograph revealed mild bone resorption at the osteotomy site. No signs of infection were found. Our laboratory results showed normal C-reactive protein (CRP) level. We encouraged him to perform partial weight bearing, and applied compressive dressing. At postoperative 3 months, he revisited our outpatient clinic on a wheelchair with similar symptoms. CRP level was normal and he was afebrile throughout the follow-up period. Radiograph revealed four broken distal fixation screws, with one screw pulled out and positioned within the knee joint. Computed Tomography (CT) revealed a widened gap and fractured lateral hinge (Fig. 2).

**Fig. 3:**
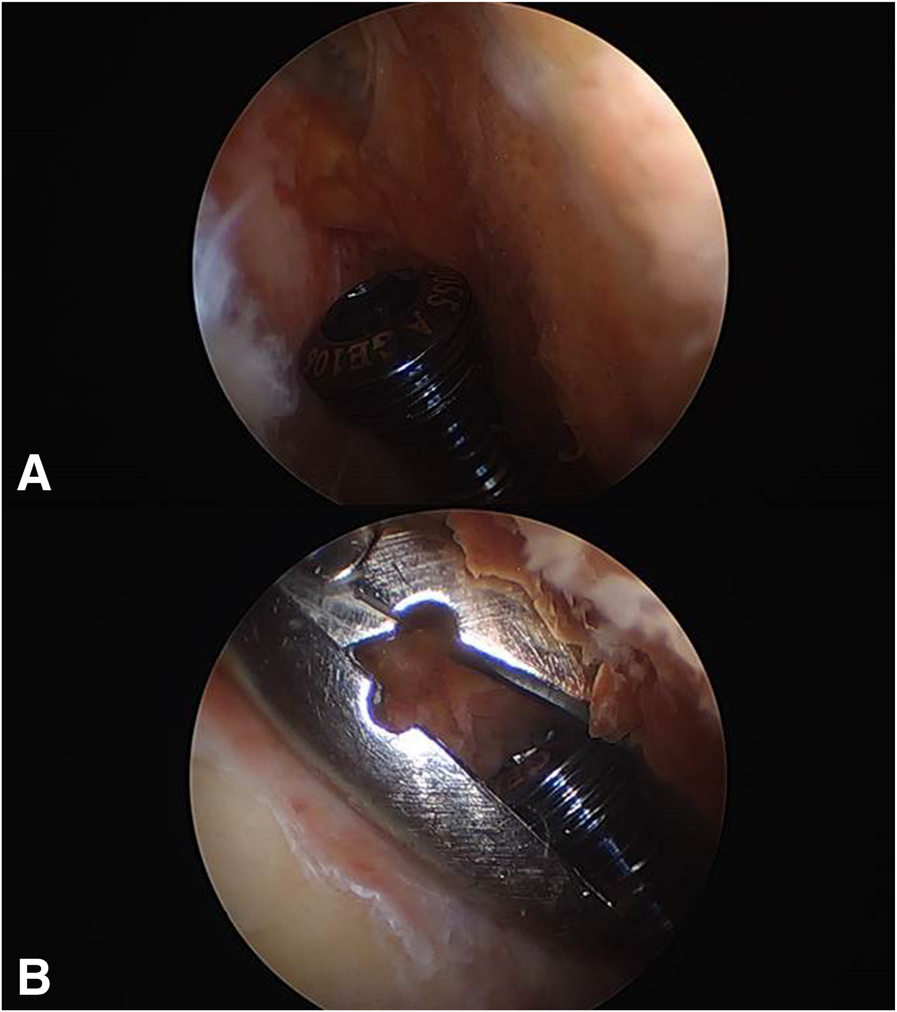
Arthroscopic image showing the removed residual portion of the broken screw: broken screw in the lateral gutter (**a**); and arthroscopic removal (**b**)

**Fig. 4:**
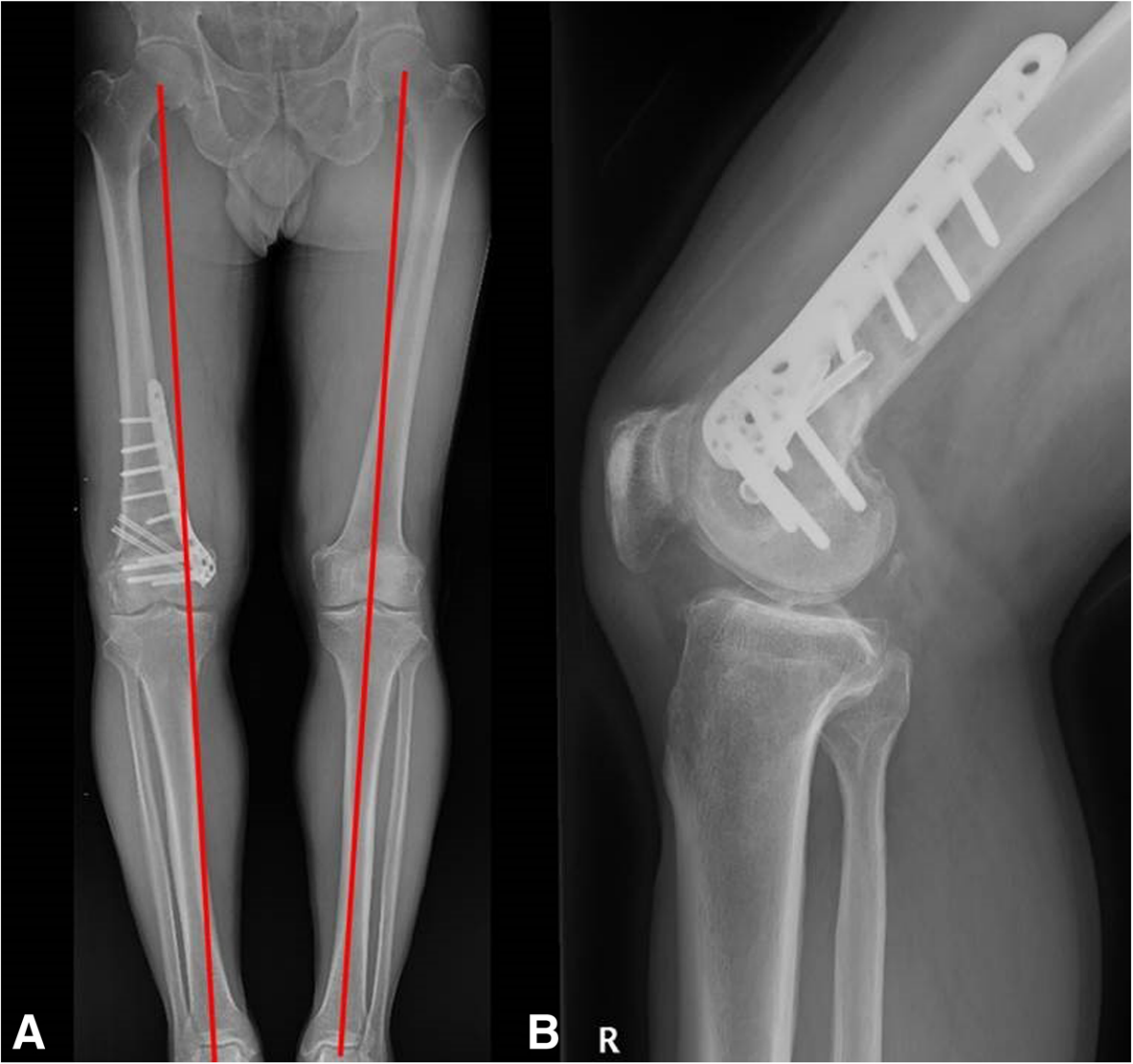
After revision DFO: restored coronal alignment (**a**); and lateral radiograph (**b**)

Re-operation was performed. The distal screws were removed first. Among the four screws, the head portions of the three screws were removed, however one screw could not be found at the previous surgical site. The tip portions of the four screws were removed using a coring reamer and the remaining hardware was removed. The osteotomy site had minimal stability. At the anterior portion of the osteotomy site, the patellofemoral joint had a connection with the operated site. The pulled-out portion of the distal screw was assumed to have moved into the joint via this path, further strengthening the reason why joint fluid was aspirated from an extra articular site. The connection was closed and arthroscopy was performed to remove the migrated screw (Fig. 3). Two cannulated lag screws were inserted between proximal-lateral and distal-medial fragments for compression of the lateral hinge. Subsequently a locking plate with a larger profile was reinserted for a more stable fixation (Fig. 4). After the re-operation, his symptoms improved and the swelling disappeared. He was satisfied with the outcome of the re-operation and union was achieved at postoperative 6 months. Informed consent was obtained from the patient for all procedures.

## Discussion and conclusions

In this report, we described a case of hardware failure after closing wedge DFO. In the analysis of the complication associated with closing and opening-wedge DFOs, both these procedures have similar major potential complications such as fracture, hematoma, and pulmonary embolism. Minor complications such as stiffness and postoperative pain also appeared in both groups. The common complications were plate prominence, discomfort, or irritation over the plate [5]. Nonunion or hardware failure is a generally a concern during opening wedge DFO due to the opened gap. However, in one systematic review, medial closing wedge DFO showed a higher incidence of hardware failure and loss of correction when compared to opening wedge DFO [6]. This implies that closing wedge DFO is not safe.

Outcome of DFO may be improved by the precise method of fixation. The DFO plate configuration is slightly anteriorly positioned, and distal fixation holes are limited when compared with conventional plates for distal femur fractures, which is curved posteriorly for fixation of the femoral condyle. This may affect fixation stability. Correct surgical indication and rehabilitation also influence the complication rate of DFO [7].

With regards to the failure mechanism in our patient, the intraoperative stability and postoperative management were similar to other patients who underwent DFO. However, this patient experienced an unexpected migration of a broken screw to the knee joint. There was recurrent painful swelling of the knee, and aspiration from the operated site revealed joint fluid.

In addition, there was atrophic nonunion with bony resorption occurring at the osteotomy site. These two occurrences do not bode well for hardware stability since hardware failure is related to mechanical instability, while bony resorption is a biological deficiency. Based on our assumption, the presence of joint fluid is an obstacle to bone union, leading to metal failure [8]. Therefore, preservation of the suprapatellar pouch during DFO is of paramount importance. In the present case, fixation was performed slightly anteriorly using a custom-made DFO plate, whereby the suprapatellar pouch might have been penetrated during the approach or during subsequent plate application [1]. This was confirmed during the revision surgery, and the broken screw was assumed to have migrated via this path.

The present study was aimed at providing a lesson to surgeons who might experience a similar situation. We assumed that the causes of the complication were as follows: Firstly, there might have been penetration of the joint during the index surgery. This could have caused regurgitation of joint fluid to the osteotomy site, impeding bone union. Secondly, metal failure was a result of nonunion, with subsequent migration of broken screw into the joint.

Postoperative knee swelling should be further investigated to ascertain the reason, as seen in this case whereby joint fluid aspirated from an extra articular operation site should raise the suspicion of an adverse cause. Finally, the most important message would be to preserve the suprapatellar pouch during DFO and to repair the capsule if breached.

DFO is performed at the adjacent area of the knee joint, and surgeons should preserve the suprapatellar pouch and knee joint to prevent such unusual complication as occurred in our case.

## Abbreviations

CRP: C-reactive protein
CT: Computed Tomography
DFO: Distal Femoral Osteotomy
